# *Ex Vivo* Nicotine Stimulation Augments the Efficacy of Human Peripheral Blood Mononuclear Cell-Derived Dendritic Cell Vaccination via Activating Akt-S6 Pathway

**DOI:** 10.1155/2015/741487

**Published:** 2015-08-13

**Authors:** Yan Yan Wang, Yi Wen Yang, Xiang You, Xiao Qian Deng, Chun Fang Hu, Cong Zhu, Jun Yao Wang, Jiao Jiao Gu, Yi Nan Wang, Qing Li, Feng Guang Gao

**Affiliations:** ^1^Department of Immunology, Basic Medicine Science, Medical College, Xiamen University, Xiamen 361102, China; ^2^The State Key Laboratory for Oncogenes and Related Genes, Shanghai Jiao Tong University, Shanghai 200032, China

## Abstract

Our previous studies showed that *α*7 nicotinic acetylcholine receptor (nAchR) agonist nicotine has stimulatory effects on murine bone marrow-derived semimature DCs, but the effect of nicotine on peripheral blood mononuclear cell- (PBMC-) derived human semimature dendritic cells (hu-imDCs) is still to be clarified. In the present study, hu-imDCs (cultured 4 days) were conferred with  *ex vivo* lower dose nicotine stimulation and the effect of nicotine on surface molecules expression, the ability of cross-presentation, DCs-mediated PBMC priming, and activated signaling pathways were determined. We could demonstrate that the treatment with nicotine resulted in increased surface molecules expression, enhanced hu-imDCs-mediated PBMC proliferation, upregulated release of IL-12 in the supernatant of cocultured DCs-PBMC, and augmented phosphorylation of Akt and ribosomal protein S6. Nicotine associated with traces of LPS efficiently enhanced endosomal translocation of internalized ovalbumin (OVA) and increased TAP-OVA colocalization. Importantly, the upregulation of nicotine-increased surface molecules upregulation was significantly abrogated by the inhibition of Akt kinase. These findings demonstrate that *ex vivo* nicotine stimulation augments hu-imDCs surface molecules expression via Akt-S6 pathway, combined with increased Ag-presentation result in augmented efficacy of DCs-mediated PBMC proliferation and Th1 polarization.

## 1. Introduction

Dendritic cells (DCs) are professional antigen-presenting cells (APCs) that recognize extracellular antigens in the peripheral tissue. On recognition of microbial substances, DCs migrate toward the draining lymph node, where they can induce antigen-specific T-cell priming and reveal therapeutic and protective antitumor immunity [1, 2]. In previous studies, we have demonstrated that* ex vivo* nicotine stimulation has stimulatory effects on murine bone marrow-derived semimature DCs (imDCs), which reveal efficient upregulation of surface molecules through *α*7 nicotinic acetylcholine receptor (nAchR) [3–6]. Further studies showed that the treatment with nicotine increases surface molecules expression via PI3K-Akt pathway and nicotine-treated murine imDCs has antitumor effects in both Lewis lung cancer and hepatocellular carcinoma [4, 5]. Although several groups have documented that nicotine has positive effects in the treatment of neurodegenerative diseases, ulcerative colitis, and Tourette syndrome [7–9], it is the first time of our studies demonstrating that nicotine-treated imDCs have preventive and therapeutic effects on murine tumor formation. However, the effect of* ex vivo* nicotine stimulation on human peripheral blood mononuclear cells- (PBMC-) derived imDCs (hu-imDCs) is still uncertain.

For T-cell activation, internalized antigens are degraded, and the resulting peptides are loaded to MHC molecules and transported to plasma membrane, where these complexes are recognized by antigen-specific T cells. While peptides which resulted from soluble antigen can be loaded to MHC class II molecules, it can also be loaded to MHC class I molecules, a process termed cross-presentation [10, 11]. Efficient cross-presentation requires antigen uptake-mediated entrance of antigen into early endosome [12], which was mediated by mannose receptor [13]. Meanwhile, the critical role of proteasome in generating epitopes and transporting internalized antigen across the endosomal membrane into cytoplasm was also documented [14]. Although previous studies revealed that* ex vivo* nicotine stimulation increases antigen internalization and promotes imDCs' cross-presentation [3–6], the exact effect of endosomal translocation of internalized antigen in nicotine-increased cross-presentation is still uncertain, nevertheless the effect of nicotine on the transporting internalized antigen to cytoplasm.

In the present study, hu-imDCs (cultured 4 days) were conferred with* ex vivo* lower dose nicotine stimulation and the effect of nicotine on surface molecules expression, the ability of cross-presentation, DCs-mediated PBMC priming, and activated signaling pathways were determined. We demonstrate that* ex vivo* nicotine stimulation results in increased surface molecules expression, enhanced hu-imDCs-mediated PBMC proliferation, upregulated the release of IL-12, increased the percentage of CD8/IFN-gamma double positive cell in cocultured DCs-PBMC, and augmented phosphorylation of Akt and ribosomal protein S6. Nicotine associated with traces of LPS efficiently enhanced endosomal translocation of internalized ovalbumin (OVA) and increased TAP-OVA colocalization. Importantly, nicotine-increased upregulation of surface molecules was significantly abrogated by the inhibition of Akt kinase. These findings demonstrate that* ex vivo* nicotine stimulation augments hu-imDCs surface molecules expression via Akt-S6 pathway, combined with increased Ag-presentation result in augmented efficacy of DCs-mediated PBMC proliferation and Th1 polarization.

## 2. Materials and Methods

### 2.1. Reagents

Reagents were purchased from the following companies: (−)-nicotine (N3876), lipopolysaccharide (LPS), *α*-bungarotoxin-tetramethylrhodamine from Bungarus multicinctus (Formosan Banded Krait) (T0195), and tubocurarine chloride were obtained from Sigma-Aldrich (St. Louis, MO, USA). Recombinant human GM-CSF and IL-4 were obtained from PeproTech (Rocky Hill, NJ, USA). Endotoxin-free EndoGrade-ovalbumin (OVA) was purchased from Hyglos GmbH (Regensburg, Germany). PI3K inhibitor LY294002 and Akt inhibitor Wortmannin were from Cayman Chemical (Ann Arbor, MI, USA). Purified anti-chicken ovalbumin (OVA) (Clone TOSGAA1) was obtained from Biolegend (San Diego, CA). Antibodies to EEA1, Rab7, and TAP were from Cell Signaling Technology (Beverly, MA, USA). RPMI-1640 medium and fetal bovine serum (FBS) were purchased from HyClone (Logan, UT, USA). BD Phosflow antibodies to phospho-p38 (p-T180/pY182) (Clone 36/p38), phospho-Erk1/2 (p-T202/pY204) (Clone 20A), phospho-Akt (pS473) (Clone M89-61), phospho-S6 (pS235/pS236) (Clone N7-548), and human IL-12 ELISA Kit were from BD Biosciences (San Jose, CA, USA). BrdU Cell Proliferation Kit was obtained from Roche (Roche Diagnostics GmbH, Germany). Fluorescence conjugated antibodies to human CD80, CD86, 4-1BBL, CD8, IFN-*γ*, MHC class I, and MHC class II molecules were obtained from eBioscience (San Diego, USA). Mounting medium for fluorescence with DAPI was obtained from Vector Laboratories, Inc. (Burlingame, CA, USA). Human PBMC isolation reagent was from Haoyang Biological Manufacture Co., Ltd. (Tianjin, China). Rab5, goat anti-mouse IgG and donkey anti-goat IgG secondary antibodies were from Abcam (New Territories, HK).

### 2.2. Generation of Human PBMC-Derived imDCs

Briefly, human PBMC was prepared from the donor's blood by gradient density centrifugation using PBMC isolation reagents and cultured at a density of 1 × 10^6^ cells/mL in RPMI 1640 medium in the presence of 100 ng/mL recombinant human GM-CSF and 100 ng/mL IL-4. Nonadherent cells were gently washed out with PBS on day 4; the remaining loosely adherent clusters were used as hu-imDCs. Cells were synchronized by serum starvation (in RPMI 1640 with 0.5% FBS) for 4~6 h before the further treatment. This study was conducted with the understanding and the consent of all the blood donors and was approved by the Institutional Review Board for Human Subjects at the Medical College of Xiamen University.

### 2.3. Human imDCs Treatment

To determine the effect of* ex vivo* nicotine stimulation on surface molecules expression, hu-imDCs were exposed to nicotine (10^−7^ mol/L) for 12 to 15 h after cell synchronization by 0.5% serum starvation. The pretreatment with 2 *μ*g/mL *α*-bungarotoxin or (4 × 10^−5^ mol/L) tubocurarine chloride 2 h prior to nicotine stimulation was conducted to investigate the role of *α*7 nAchR in nicotine-increased DCs' function. To elucidate the mechanism of nicotine-increased surface molecules expression, hu-imDCs were conferred with LY294002 (10 *μ*mol/L) or Wortmannin (10 *μ*mol/L) 2 h prior to nicotine (10^−7^ mol/L) exposure.

### 2.4. Flow Cytometric Measurement for Surface Molecules

The expression of surface molecules in hu-imDCs was determined by flow cytometry. Briefly, hu-imDCs were preincubated with 0.5 *μ*g CD16/CD32 antibodies for 10 min. Then, aliquot cell suspension was stained with combined primary antibody at a concentration of 1 *μ*g per 1 × 10^6^ cells. Staining was performed on ice for 30 min and cells were washed with ice-cold PBS, containing 0.1% NaN3 and 0.5% BSA. Flow cytometry was done with FACSCalibur and data were analyzed with CellQuest software.

### 2.5. Flow Cytometric Measurement for Intracellular Signaling Molecules

To determine the effect of nicotine on kinases phosphorylation, hu-imDCs were collected by trypsination and treated with nicotine (10^−7^ mol/L) for 15 min. The phosphorylation of related kinase was determined by BD Phosflow. Briefly, at the end of nicotine treatment, the cells were immediately mixed with warmed BD Phosflow Fix Buffer I and incubated at 37°C for 10 min. Then, the cells were washed with BD Pharmingen Stain Buffer and permeabilized by incubation with cold BD Phosflow Perm Buffer III for 30 min on ice. After complete washes, the cells were stained with BD Phosflow antibodies and flow cytometry was done with FACSCalibur and data were analyzed with CellQuest software.

### 2.6. Confocal Microscope Analyses

The effect of nicotine on the endosomal translocation of antigen and the complex formation of ovalbumin-derived peptide-MHC class I and II molecules were investigated by confocal microscope analyses. Briefly, hu-imDCs were exposed to nicotine (10^−7^ mol/L) for 12~15 h. Then, the cells were conferred with endotoxin-free EndoGrade-ovalbumin (50 *μ*g/mL) 60 min pulse with or without short period (20 min) LPS (1 ng/mL) stimulation. After that, the DCs were fixed and permeabilized in 100% methanol for 15 min, washed with PBS, and blocked with 10% nonfat milk for 3 h. Primary antibodies were incubated in a humid chamber overnight at 4°C. Finally, fluorescence-conjugated secondary antibodies were incubated for 1 h at 37°C. DAPI counterstaining was performed to visualize cell nuclei. The cell was washed three times in each step to remove nonbinding substance and images were recorded by a confocal fluorescence microscope at the wavelength of 488 nm.

### 2.7. Ag-Specific PBMC Proliferation and CTL Priming Assay

Briefly, hu-imDCs (cultured for 4 d) were pretreated with *α*-bungarotoxin (2 *μ*g/mL) or tubocurarine chloride (4 × 10^−5^ mol/L) 2 h prior to nicotine treatment. Then, the DCs were conferred with 3 h endotoxin-free EndoGrade-ovalbumin (50 *μ*g/mL) pulse with 60 min LPS (1 ng/mL) stimulation. Responder cells were prepared from PBMC of the same donor. Stimulator cells were mixed with responders at a ratio of 1 : 1. After 5 d of coculture, Ag-specific PBMC proliferation was determined by BrdU cell proliferation assay. To determine CTL priming, the proliferated PBMC cells were performed intracellular IFN-gamma and CD8 positive flow cytometric analyses.

### 2.8. Quantification of IL-12 Production

The release of IL-12 in the supernatant of MLR was determined by ELISA according to the standard procedure. Briefly, plates were treated with coating antibody at 4°C overnight, washed with PBS, and blocked with assay buffer at room temperature for 2 h. The blocked plates were washed twice with thorough aspiration of microwell contents between washes. After the last wash step, empty wells and tap microwell strip to remove excess wash buffer. Samples of assay buffer, biotin-conjugated detector antibodies were added to microwells and the plates were incubated at room temperature for 2 h. After incubation, plates were washed 5 times and added with streptavidin-HRP. After 1 h of incubation at room temperature, plates were washed and TMB substrate solution was added to all wells for color formation. Plates were incubated at room temperature and stop solution was added to stop the enzyme reaction at appropriate time. Absorbance of each microwell was read using 450 nm as primary wave length and 620 nm as reference wavelength, respectively.

### 2.9. Western Blot

For analysis of Akt and S6 phosphorylation regulated by nicotine on hu-imDCs, proteins were obtained in lysis buffer. Protein lysates (30 *μ*g/mL) were electrophoresed on 10% SDS-PAGE gels, transferred to PVDF membranes, and blotted with phospho-Akt and phospho-S6 antibodies, followed by anti-mouse horseradish peroxidase and detection by chemiluminescence ECL. As loading controls, antibody against *β*-actin was used.

### 2.10. Statistical Analysis

All data were expressed as mean and standard error means. Statistical significance was tested using the Student *t*-test or one-way ANOVA with post-Newman-Keuls test. Statistical differences were considered to be significant if *P* < 0.05.

## 3. Results

### 3.1. Nicotine Upregulates Surface Molecules Expression in Human Semimature DCs

Human immature monocyte-derived DCs are commonly generated by culturing adherent peripheral monocytes with GM-CSF and IL-4 for up to 6 days [15–17]. Meanwhile, in a 4-day culture system, murine semimature DCs could differentiate into a regulatory DCs subset by splenic stromal cells [18] and have potential antitumor effects [3–6]. To explore the effect of nicotine on DCs' maturation and viability, hu-imDCs (cultured 4 days) were conferred with* ex vivo* nicotine stimulation. Consistent with murine semimature DCs' results [19, 20], 10^−7^ mol/L nicotine has no effect on cell viability of hu-imDCs (see Supplementary Figure 1 in Supplementary Material available online at http://dx.doi.org/10.1155/2015/741487). While 83.43% vehicle-treated hu-imDCs expressed human DCs specific marker CD1a, the treatment with nicotine increased the level of CD1a to about 150% (Supplementary Figure 2a). The analyses of CD11c expression also achieved the similar results (Supplementary Figure 2b). Considering* ex vivo* lower dose nicotine stimulation promotes the development of mouse semimature DCs [20, 21], the 4-day culture system might be a specific condition for the effect of lower dose nicotine stimulation on hu-imDCs.

**Figure 1:**
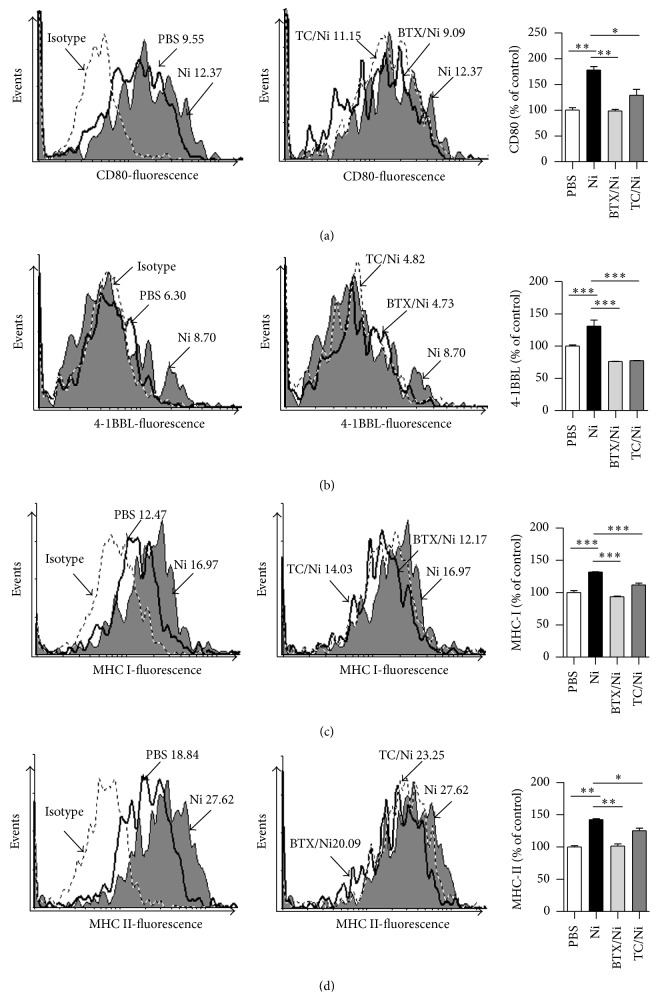
Nicotine upregulates surface molecules expression in human semimature DCs. (a–d) DCs derived from human PBMC with 100 ng/mL recombinant human GM-CSF and IL-4 were conferred with *α*-bungarotoxin (2 *μ*g/mL) or tubocurarine chloride (4 × 10^−5^ mol/L) 2 h prior to nicotine (10^−7^ mol/L) 12~15 h stimulation. The effect of nicotine on the expression of CD80 (a), 4-1BBL (b), MHC class I (c), and MHC class II (d) molecules was determined by flow cytometry. Numbers in histogram indicated mean fluorescence intensity (MFI) of test samples. Data were given as mean ± SEM, one-way ANOVA with post-Newman-Keuls test. ^*∗*^ *P* < 0.05, ^*∗∗*^ *P* < 0.01, and ^*∗∗∗*^ *P* < 0.001. One representative from 3 independent experiments is shown. Ni: nicotine; BTX: *α*-bungarotoxin; and TC: tubocurarine chloride.

MHC class I and II molecules are the components of antigenic peptide-MHC complex for antigen presentation [10–14]. While CD80/CD86 are important costimulatory molecules in T-cell-APC interaction [2, 22], 4-1BBL, which sends signals to 4-1BB-expressing cells [23, 24], was found to play critical roles in preventing activation-induced cell death [25, 26]. To explore the exact effect of nicotine stimulation on the expression of surface molecules, hu-imDCs were conferred with nicotine exposure and the expression of CD80, 4-1BBL, and MHC class I and II molecules was determined by flow cytometry. Compared with vehicle-treated cells, the treatment with nicotine obviously increased the expression of CD80, 4-1BBL, revealing about 177% and 131% upregulation, respectively (Figures 1(a) and 1(b)). The determination of MHC class I and II molecules also achieved similar results (Figures 1(c) and 1(d)). Interestingly, when dot plot of flow cytometry was used to investigate the effect of nicotine on DCs maturation, the treatment with nicotine not only increased CD1a-CD11c double positive DCs' percentage (Supplementary Figure 3a) but also augmented MHC class II-CD86 double positive cell population (Supplementary Figure 3b). These data indicate that* ex vivo* nicotine stimulation not only increases the expression of CD80 and 4-1BBL but also upregulates the expression of MHC class I and II molecules in hu-imDCs.

*α*7 nAchR, which is mainly expressed in DCs, is involved in nicotine-augmented expression of surface molecules in murine semimature DCs [19]. To investigate the potential roles of *α*7 nAchR in nicotine-increased surface molecules expression, hu-imDCs were treated with *α*7 nAchR specific *α*-bungarotoxin or nonspecific antagonist tubocurarine chloride prior to nicotine stimulation. Despite the expression of CD80, 4-1BBL, and MHC class I and II molecules obviously upregulated by the treatment with nicotine, the usage of *α*-bungarotoxin and tubocurarine chloride efficiently abrogated nicotine's effect on surface molecules expression (Figure 1), indicating that nicotine increasing surface molecules expression is *α*7 nAchR-dependent.

### 3.2. *Ex Vivo* Nicotine Stimulation Augments Semimature DCs-Mediated PBMC Proliferation and Promotes the Release of IL-12 in the Supernatant of Cocultured DCs-PBMC

**Figure 2:**
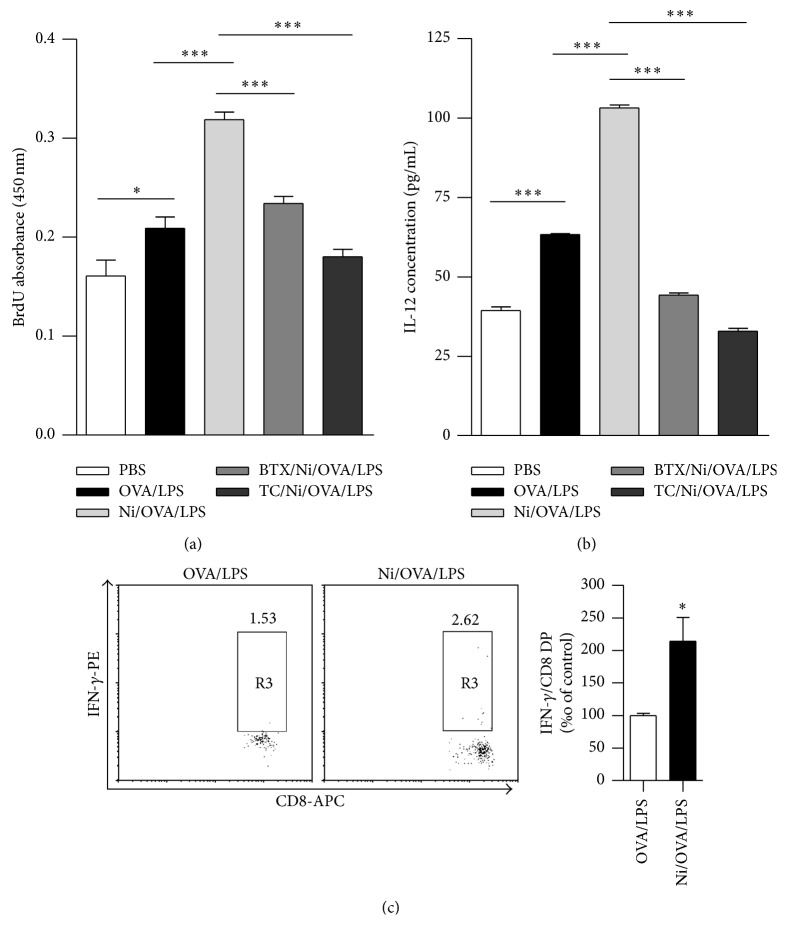
*Ex vivo* nicotine stimulation augments semimature DCs-mediated PBMC proliferation, promotes the release of IL-12, and increases the percentage of IFN-gamma/CD8 cells in the supernatant of cocultured DCs-PBMC. DCs derived from human PBMC with 100 ng/mL recombinant human GM-CSF and IL-4 were conferred with *α*-bungarotoxin (2 *μ*g/mL) or tubocurarine chloride (4 × 10^−5^ mol/L) 2 h prior to nicotine (10^−7^ mol/L) 12~15 h stimulation. Then, the DCs were pulsed with endotoxin-free EndoGrade-ovalbumin (50 *µ*g/mL) for 3 h, followed by 1 h LPS (1 ng/mL) stimulation. After that, the DCs were coincubated with PBMC of the same donor at a ratio of 1 : 1 for 5 d and the effect of nicotine on DCs-mediated PBMC proliferation, the IL-12 release in the supernatant, and IFN-gamma/CD8 cell percentage of cocultured DCs-PBMC were determined by BrdU cell proliferation assay (a), ELISA (b), and flow cytometry (c), respectively. Data were given as mean ± SEM, *n* = 2, one-way ANOVA with post-Newman-Keuls test. ^*∗*^ *P* < 0.05, and ^*∗∗∗*^ *P* < 0.001. One representative from 3 independent experiments is shown. Ni: nicotine; BTX: *α*-bungarotoxin; and TC: tubocurarine chloride.

Our previous studies showed that the treatment with lower dose nicotine promotes mouse semimature DCs-mediated cross priming [3–6]. Since* ex vivo* nicotine stimulation increases the expression of surface molecules in hu-imDCs (Figure 1, Supplementary Figure 3), we accessed the exact effect of nicotine stimulation on hu-imDCs-mediated PBMC proliferation and Th1 polarization by cocultured* ex vivo* nicotine-stimulated, ovalbumin-loaded hu-imDCs with PBMC. As endotoxins increase antigen processing in both MHC class II–restricted antigen presentation and intracellular mechanisms of cross-presentation [27, 28], we mimic the ordinary antigen by giving traces of LPS (1 ng/mL) to endotoxin-free EndoGrade-ovalbumin. Compared with controls, while the loading with endotoxin-free ovalbumin and LPS promotes hu-imDCs-mediated PBMC proliferation,* ex vivo* nicotine stimulation efficiently augmented the ability of hu-imDCs-dependent PBMC proliferation (*P* < 0.001). The pretreatment with *α*-bungarotoxin or tubocurarine chloride dramatically abolished nicotine-increased hu-imDCs mediated PBMC proliferation, which revealed about 26.65% and 43.26% inhibitory rates, respectively, (Figure 2(a)), indicating that nicotine exposure-increased hu-imDCs-mediated PBMC proliferation is *α*7 nAchR-dependent.

IL-12, an indicator of antigen-specific CTL priming, can polarize Th0 to Th1 transition and subsequently induce antivirus and antitumor immune responses [29]. Since the treatment with nicotine increased hu-imDCs-mediated PBMC proliferation, we further explored if such stimulation could polarize Th0 to Th1 transition by the IL-12 determination in the supernatant of cocultured DCs-PBMC. Compared with controls, the pulse of endotoxin-free ovalbumin with traces of LPS obviously augmented IL-12 release in the supernatant.* Ex vivo* nicotine stimulation efficiently increased IL-12 release to about 167%. The pretreatment with *α*-bungarotoxin or tubocurarine chloride obviously abolished nicotine-increased IL-12 secretion with 58%~70% inhibitory rate (Figure 2(b)). Flow cytometric measurement of intracellular IFN-gamma showed that the percentage of double positive cell of IFN-gamma and CD8 was also increased by the treatment with* ex vivo* nicotine stimulation (Figure 2(c)). These data indicate that the treatment with nicotine facilitates Th1 polarization and CTL priming in DCs-mediated PBMC proliferation.

### 3.3. The Exposure of Traces of LPS Increases the Endosomal Translocation of Antigen and TAP-OVA-MHC Class I Molecules Complex Formation in Human Semimature DCs

**Figure 3:**
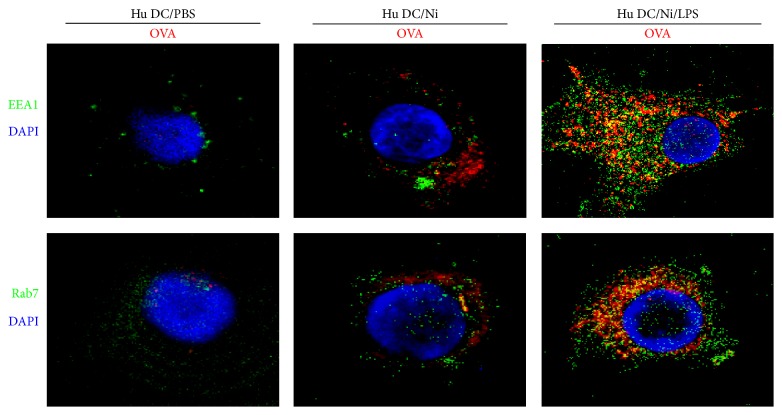
The exposure of traces of LPS increases the endosomal translocation of antigen in human semimature DCs. DCs derived from human PBMC with recombinant human GM-CSF and IL-4 were conferred with nicotine (10^−7^ mol/L) 12~15 h stimulation. Then, the DCs were exposed to 60 min endotoxin-free EndoGrade-ovalbumin (50 *μ*g/mL) pulse and 20 min LPS (1 ng/mL) stimulation. The effect of nicotine and LPS on endosomal translocation of antigen was observed by confocal microscope analyses. One representative from 3 independent experiments is shown. Ni: nicotine.

Mannose receptor, which is expressed in human DCs, uptakes antigen and determines subcellular antigen localization [31]. The localization of model antigen ovalbumin in early endosomal compartment [13] did not further mature early endosome into lysosomes but promote antigen for cross-presentation [12]. Meanwhile, microbial products such as LPS were demonstrated to trigger a program of DCs maturation which enables DCs to activate T cells [14]. To elucidate the role of LPS in nicotine-increased antigen early endosomal translocation, we pulsed hu-imDCs briefly (60 min) with endotoxin-free EndoGrade-ovalbumin, concurrently either with short period (20 min) LPS (1 ng/mL) stimulation or for the same length of time but without LPS exposure (Figure 3). We assessed endosomal translocation of antigen by ovalbumin and EEA1/Rab7 antibodies staining. Coadministration of endotoxin-free EndoGrade-ovalbumin with short period LPS exposure resulted in significantly enhanced translocation of ovalbumin to endosomal compartment (Figure 3). As efficient cross-presentation requires the entrance of antigen into a specific intracellular pathway [12], the increased endosomal translocation of ovalbumin by lower dose LPS indicates that nicotine-increased cross-presentation needs mannose receptor-mediated endosomal translocation of internalized antigen.

**Figure 4:**
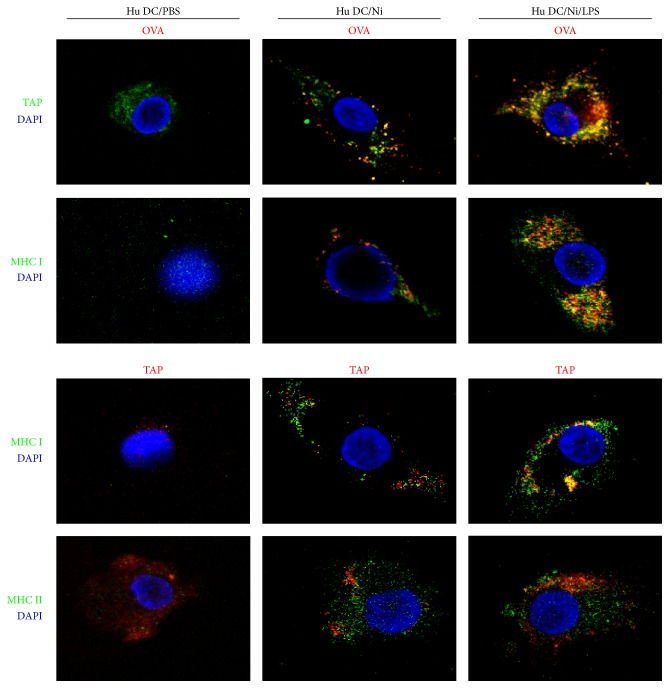
The exposure of traces of LPS increases TAP-OVA-MHC class I molecules complex formation in human semimature DCs. DCs derived from human PBMC with recombinant human GM-CSF and IL-4 were conferred with nicotine (10^−7^ mol/L) 12~15 h stimulation. Then, the DCs were exposed to 60 min endotoxin-free EndoGrade-ovalbumin (50 *μ*g/mL) pulse and 20 min LPS (1 ng/mL) stimulation. The effect of nicotine and LPS on the complex formation of TAP, OVA, and MHC class I molecules was observed by confocal microscope analyses. One representative from 3 independent experiments is shown. Ni: nicotine.

While antigens internalization via mannose receptor-mediated endocytosis was routed into a distinct murine endosomal subset [10, 11], these endosomal subsets could reimport proteasome-derived peptides into the same endosomal compartment by LPS-inducing endosomal translocation of TAP, thereafter loading these peptides onto MHC I molecules [12]. To explore the role of LPS-inducing signaling in TAP-mediated antigenic transport, we assessed the translocation of antigen, TAP, and MHC class I and II molecules in lower dose LPS (1 ng/mL) presenting condition with related antibodies staining. Compared with nicotine-treated hu-imDCs, the signaling induced by LPS administration resulted in significantly enhanced translocation of OVA-TAP and OVA-MHC class I molecules (Figure 4). The colocalization of TAP-MHC class I molecules was also augmented by the treatment with LPS, whereas TAP-MHC class II molecules colocalization was not affected anymore (Figure 4). These results indicate that nicotine-increased cross-presentation needs LPS-induced endosomal recruitment of TAP.

### 3.4. The Treatment with Nicotine Induces Akt-S6 Pathway Activation in Human Semimature DCs

**Figure 5:**
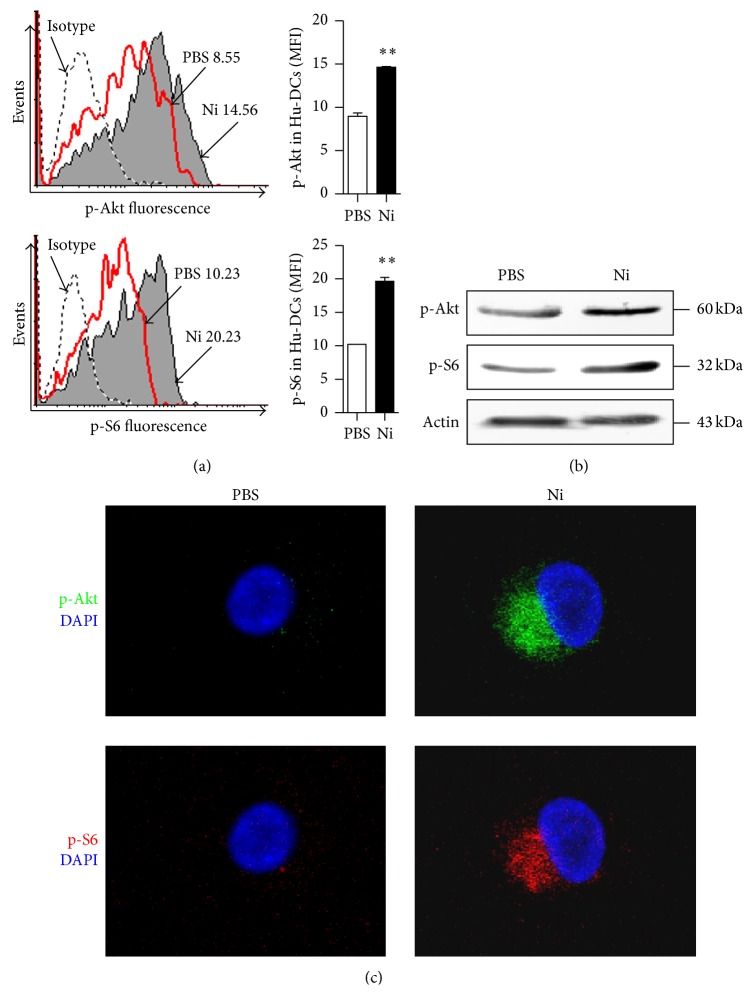
The treatment with nicotine induces Akt-S6 pathway activation in human semimature DCs. (a–c) Human PBMC-derived semimature DCs were stimulated with nicotine (10^−7^ mol/L) for 15 min and the phosphorylation of Akt and ribosomal protein S6 was determined by flow cytometry (a), Western blotting (b), and confocal microscope analyses (c), respectively. One representative from 3 independent experiments is shown. Numbers in histogram indicated mean fluorescence intensity (MFI) of test samples. Data were given as mean ± SEM, Student *t*-test, ^*∗∗*^ *P* < 0.01. Ni: nicotine.

It was reported that the Erk1/2-p38-JNK MAPK and PI3K-Akt pathways could be activated by the treatment with nicotine and play important roles in nicotine-augmented surface molecules expression in murine DCs [5, 6, 19]. Meanwhile, the activation of Akt-mTORC1 induced by nicotine promotes structural plasticity in mesencephalic dopaminergic neurons was also documented [32]. To explore the role of Akt-mTOR in nicotine-increased DCs surface molecules expression, we thereafter tested the effect of nicotine on Erk1/2-p38 MAPK and Akt-S6 pathway activation. Unlike traditional methods such as Western blotting, intracellular phosphoprofiling could examine cellular subpopulations in complex samples and analyze phosphoprotein signaling in rare cell subtypes [33, 34]. While the phosphorylation of Erk1/2 and p38 could be obviously augmented by nicotine stimulation (Supplementary Figure 4), the treatment with nicotine rapidly increased the phosphorylation status of Akt and S6 in the early 15 minutes, which revealed 163.5% and 191.8% increase, respectively, by flow cytometry assay (Figure 5(a)). Western blotting (Figure 5(b)) and confocal microscope analyses (Figure 5(c)) also showed that Akt-S6 pathway could be activated by the treatment with nicotine, indicating that Erk1/2-p38 and Akt-S6 pathways might be play potential roles in nicotine-increased surface molecules expression.

### 3.5. Nicotine Increases Surface Molecule Expression in Human Semimature DCs by Activating Akt-S6 Pathway

**Figure 6:**
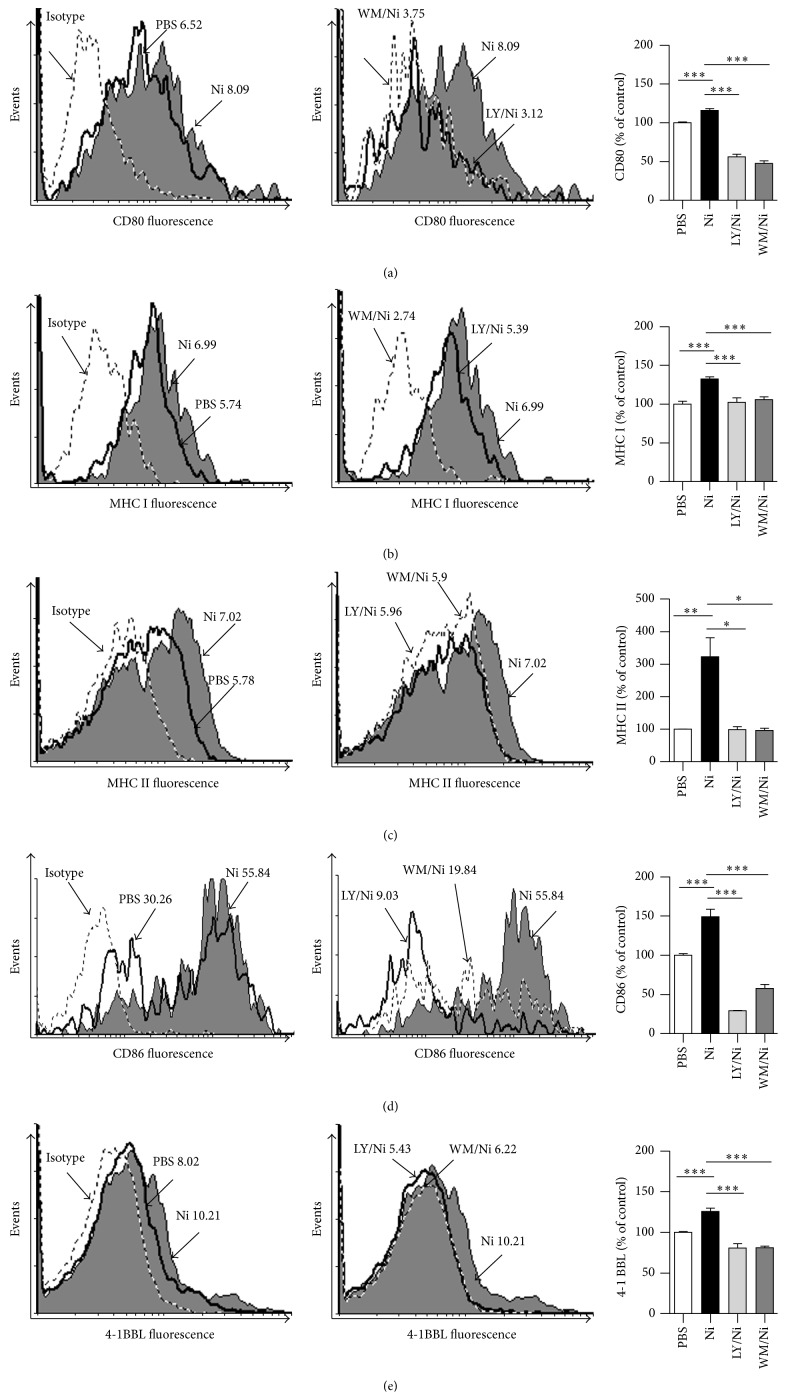
Nicotine increases surface molecule expression in human semimature DCs by activating Akt-S6 pathway. (a–e) DCs derived from human PBMC with human GM-CSF and IL-4 were conferred with LY294002 (10 *μ*mol/L) or Wortmannin (10 *μ*mol/L) 2 h prior to 12~15 h nicotine (10^−7^ mol/L) stimulation. The effect of Akt inhibition on nicotine-increased expression of CD80 (a), MHC class I (b), MHC class II (c), CD86 (d), and 4-1BBL (e) was determined by flow cytometry. Numbers in histogram indicated mean fluorescence intensity (MFI) of test samples. Data were given as mean ± SEM, one-way ANOVA with post Newman-Keuls test. ^*∗*^ *P* < 0.05, ^*∗∗*^ *P* < 0.01, and ^*∗∗∗*^ *P* < 0.001. One representative from 3 independent experiments is shown. Ni: nicotine; LY: LY294002; WM (Wort): Wortmannin.

Despite the treatment with nicotine activates Akt-S6 pathway, the exact role of Akt-S6 in nicotine-increased surface molecule expression in hu-imDCs is still uncertain. We accessed the expression of surface molecules in human DCs by inhibition relevant kinases. Compared with vehicle-treated cells, the treatment with nicotine increased about 162.94%, 216.4%, 322.4%, 149%, and 126% expression of CD80, MHC class I, MHC class II, CD86, and 4-1BBL, respectively (Figure 6). Importantly, in contrast to the treatment with nicotine, the pretreatment with both Akt inhibitor LY294002 and Wortmannin obviously abolished nicotine's effect on these surface molecules upregulation (Figure 6). All these results indicate that the activation of Akt-S6 is involved in nicotine-increased surface molecules expression in hu-imDCs.

## 4. Discussion

In the present study, we investigated the effect of nicotine on surface molecules expression, the ability of cross-presentation, DCs-mediated PBMC priming, and activated signaling pathways by exposing hu-imDCs to nicotine stimulation. We demonstrate that the upregulation of surface molecules, the enhancement of hu-imDCs-mediated PBMC proliferation, and increased release of IL-12 in the supernatant of cocultured DCs-PBMC are *α*7 nAchR-dependent. Importantly, the percentage of CD8/IFN-gamma double positive cell was increased by the treatment with* ex vivo* nicotine stimulation. Moreover, the phosphorylation of Akt and ribosomal protein S6 induced by nicotine stimulation play vital roles in nicotine-increased surface molecules expression. Interestingly, the endosomal translocation of internalized ovalbumin and increased TAP-OVA colocalization are also augmented by the treatment with traces of LPS.

Nicotine, a major component of cigarette smoke which promotes established tumor metastasis and increases overall mortality in cancer patients [7], is widely accepted as a risk factor for atherosclerosis [19]. Since the nAchR is mainly expressed in neuron and affects neurodegenerative disease progression [35], the effects of nicotine on promoting lung cancer development, reducing the efficacy of chemotherapeutic agents [8], and activating hypoxia-inducible factor-1 *α* expression [9] can not exclude the potential roles of nAchR in regulating the function of DCs and neuron. Recently, *α*7 nAchR has been documented to exist in murine DCs and play pivotal roles in regulating DCs' function [3–6, 22]. In response to Th2-promoting stimuli, both mouse and human DCs generated to 6-7 days in the presence of the immune modulator nicotine (nicDCs) preferentially support the differentiation of antigen-specific IL-4-producing Th2 effector cells [15]. Furthermore, NicDCs could produce lower levels of proinflammatory cytokines when compared with DCs differentiated in the absence of nicotine [16, 17], indicating the modulating role of nicotine in DCs' development. Hence, the exact effect of nicotine on DCs' function is currently contradictory. Using splenic stromal cells to mimic the immune microenvironment, murine immature DCs (cultured 4 days) could induce their differentiation into a new regulatory DC subset by both stromal cell contact and stromal cell-derived transforming growth factor-beta [18]. In our previous studies, murine immature DCs (cultured 4 days) have been demonstrated to reveal potential antitumor effects by* ex vivo* nicotine stimulation [3–6]. Further studies reveal that although the treatment with nicotine and LPS upregulates surface molecules expression [20] and enables DCs to present Ags in the context of MHC I molecules, the CD8^+^ T-cell priming is refractory [36]. As the biological effect of nicotine on lymphocyte is dependent on dose of nicotine, the duration of exposure[37], and the LPS existence in experiment system [20], the controversy of nicotine's effect on DCs might be attributed to the differences of experimental design, species, duration of exposure, and especially the nicotine concentration used in these experiments. With the treatment of 200 mg/mL nicotine, Nouri-Shirazi and Guinet found that the exposure to nicotine adversely affects the dendritic cell system and compromises host response to vaccination [38]. On the other hand, the presence of nicotine (0–200 *μ*g/mL) promotes the development of mouse DC precursors into a semimature phenotype and supports the differentiation of ovalbumin- (OVA-) specific naïve T cells into effector memory cells [21]. In our systematic studies, 10^−7^ mol/L nicotine (16.5 ng/mL) was used as *α*7 nAchR agonist to stimulate human or murine semimature DCs (cultured 4 days). Hence, lower dose nicotine stimulation (16.5 ng/mL) and DCs with a semimature phenotype were the specific conditions for achieving increased DCs vaccination. It does not implicate that nicotine itself has the similar biological effect on cancer growth.

Nicotine increasing mouse immature DCs-mediated cross priming could be abolished by *α*7 nAchR specific antagonist *α*-bungarotoxin and nonspecific antagonist tubocurarine chloride [3–6], indicating the vital role of *α*7 nAchR in nicotine-augmented murine DCs' function. In the present study, hu-imDCs were pretreated with *α*-bungarotoxin or tubocurarine chloride prior to* ex vivo* nicotine stimulation to investigate the role of *α*7 nAchR in nicotine-augmented hu-imDCs' function. Consistent with the finding of murine DCs, not only the upregulation of surface molecules (Figure 1) but also DCs-mediated PBMC priming (Figure 2) was abrogated by the blockage of *α*7 nAchR. These data indicate that nicotine-increased DCs-dependent PBMC proliferation and surface molecules upregulation are also *α*7 nAchR-dependent.

Antigens internalized by DCs via fluid phage pinocytosis or scavenger receptor-mediated endocytosis are rapidly targeted toward lysosomal structures, where they are degraded instantly and processed for presentation on MHC II molecules [39]. Meanwhile, antigens, which are internalized by the mannose receptor, are routed into endosome subpopulation. Antigens of endosome compartment are protected from lysosomal degradation and are processed exlusively for cross-presentation [12]. Recently, endotoxin was found to increase recruitment of TAP toward antigen-containing endosomes and enable the retranslocation of proteasome-derived peptides into the same endosome subset [12, 27, 28]. In this study, compared with endotoxin-free ovalbumin loading, the pulse of endotoxin-free ovalbumin with traces of LPS not only increased the endosomal translocation of antigen (Figure 3) but also augmented the formation of OVA-TAP-MHC class I complex (Figure 4), indicating that nicotine-enhanced hu-imDCs cross-presentation might also attribute to LPS-increased endosomal protection from lysosomal degradation and the endosomal recruitment of TAP.

Akt, which could be phosphorylated within the activation loop at threonine 308 and the C-terminus at serine 473, promotes cell survival by inhibiting proapoptotic function of Bad [40]. We have demonstrated that nicotine activates PI3K-Akt pathway and upregulates CD80 molecules expression in murine DCs [5, 6]. Jossin and Goffinet's study also showed that PI3K-Akt signal controls cortical development and regulates dendritic growth [41]. In the present study, Akt pathway was found to play vital role in nicotine-increased surface molecules expression in hu-imDCs (Figure 6). Ribosomal protein S6, which plays a role in regulating the translation of RNAs and thus controlling the growth and proliferation of cells [42], was efficient phosphorylated by the treatment with nicotine (Figure 5). Although mTORC1 was involved in nicotine-induced structural plasticity in mesencephalic dopaminergic neurons [32], the phosphorylation of S6 ribosomal protein was reported to upregulate ribosomal translocation of RNA species coding for other ribosomal proteins, peptide elongation factors [43]. Hence, the exact role of S6 ribosomal protein in nicotine-regulated surface molecules expression in human DCs still needs further investigation.

In conclusion, all the data presented here indicate that nicotine upregulating surface molecules and enhancing DCs-mediated PBMC proliferation and the release of IL-12 are *α*7 nAchR-dependent. Nicotine-enhanced hu-imDCs cross-presentation attributes to LPS-increased endosomal translocation of internalized ovalbumin and the endosomal recruitment of TAP.

## Supplementary Material

Figure S1: The treatment with lower dose nicotine has no effect on cell viability of human semi-mature DCs.
Figure S2: Nicotine up-regulates CD11c and CD1a expression in human semi-mature DCs.
Figure S3: The treatment with nicotine promotes human DCs maturation in human semi-mature DCs.
Figure S4: The treatment with nicotine induces Erk-p38 activation in human semi-mature DCs.

## Abbreviations

DCs: Dendritic cells
nAChR: Nicotinic acetylcholine receptor
S6: Ribosomal protein S6
PI3K: Phosphatidylinositol-3-kinase
Ni: Nicotine
MLRs: Mixed lymphocyte reactions
LPS: Lipopolysaccharide
PBMC: Peripheral blood monouclear cells
EEA1: Early endosome antigen 1
TAP: Transporter associated with antigen processing
Rab1: Ras-Related superfamily of guanine nucleotide binding proteins 1.
